# Advances in the Development of Antiviral Strategies against Parvovirus B19

**DOI:** 10.3390/v11070659

**Published:** 2019-07-18

**Authors:** Elisabetta Manaresi, Giorgio Gallinella

**Affiliations:** Department of Pharmacy and Biotechnology, University of Bologna, I-40138 Bologna, Italy

**Keywords:** parvovirus B19, erythroid progenitor cells, antiviral compounds, intravenous immunoglobulin (IVIG), hydroxyurea, cidofovir, brincidofovir, coumarin derivatives, flavonoids

## Abstract

Parvovirus B19 (B19V) is a human pathogenic virus, responsible for an ample range of clinical manifestations. Infections are usually mild, self-limiting, and controlled by the development of a specific immune response, but in many cases clinical situations can be more complex and require therapy. Presently available treatments are only supportive, symptomatic, or unspecific, such as administration of intravenous immunoglobulins, and often of limited efficacy. The development of antiviral strategies against B19V should be considered of highest relevance for increasing the available options for more specific and effective therapeutic treatments. This field of research has been explored in recent years, registering some achievements as well as interesting future perspectives. In addition to immunoglobulins, some compounds have been shown to possess inhibitory activity against B19V. Hydroxyurea is an antiproliferative drug used in the treatment of sickle-cell disease that also possesses inhibitory activity against B19V. The nucleotide analogues Cidofovir and its lipid conjugate Brincidofovir are broad-range antivirals mostly active against dsDNA viruses, which showed an antiviral activity also against B19V. Newly synthesized coumarin derivatives offer possibilities for the development of molecules with antiviral activity. Identification of some flavonoid molecules, with direct inhibitory activity against the viral non-structural (NS) protein, indicates a possible line of development for direct antiviral agents. Continuing research in the field, leading to better knowledge of the viral lifecycle and a precise understanding of virus–cell interactions, will offer novel opportunities for developing more efficient, targeted antiviral agents, which can be translated into available therapeutic options.

## 1. Introduction

Parvovirus B19 (B19V), a single-stranded DNA virus in the family *Parvoviridae* [1], is a human pathogenic virus, characterized by a selective but not exclusive tropism for erythroid progenitor cells. Globally diffuse, it is responsible for an ample range of clinical manifestations, whose characteristics and outcomes depend on the interplay between the viral properties as well as the physiological and immune status of the infected individuals. The clinical attitude towards B19V infection is normally conservative, in the idea that consequences of infections are mild, self-limiting, and controlled by the development of a specific immune response. However, clinical situations can be more complex, depending on the genetic or physiological background of the host, in the case of underlying diseases or inefficiency of the immune response, and in the evenience of maternal transmission to fetus. Thus, in many situations clinical care is needed, relying on the currently available treatments that are only supportive, symptomatic, or unspecific, and in many cases of limited efficacy. Research aimed at the development of antiviral strategies should therefore be considered of highest importance for increasing the available options for more specific and effective therapeutic treatments. This field of research has been explored in recent years, and a few published works already report some achievements as well as interesting future perspectives.

## 2. B19V Structure

**Figure 1:**
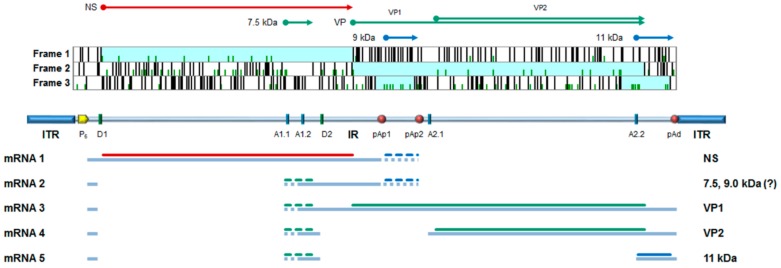
B19V genome organization. Top: major open reading frames identified in the positive strand of genome; arrows indicate the coding sequences for the viral proteins. NS, non-structural protein; VP, structural proteins, colinear VP1 and VP2, assembled in a T = 1 icosahedral capsid; and 7.5 kDa, 9.0 kDa, and 11 kDa: minor non-structural proteins. Center: a schematic diagram of B19V genome indicating the two inverted terminal regions (ITR), and the internal region (IR) with the distribution of cis-acting functional sites (P6, promoter; pAp1, pAp2, proximal cleavage-polyadenylation sites; pAd, distal cleavage-polyadenylation site; D1 and D2, splice donor sites; A1.1, A1.2, A2.1, and A2.2, splice acceptor sites). Bottom: simplified transcription map of B19V genome, indicating the five classes of mRNAs (mRNA 1–5) with respective alternative splicing/cleavage forms (dashed), and their coding potential. Adapted from Reference [5].

B19V shares genetic and structural features common to the family (comprehensively reviewed in [2,3]). The genome, a linear ssDNA molecule of 5.6 kb, is organized in a unique internal region, containing all the coding sequences, flanked by inverted terminal regions that serve as origins of replication (ORF). In its internal region, the genome presents two major ORFs, in the left side for the non-structural protein (NS), and in the right side for the two colinear capsid proteins, VP1 and VP2. Minor ORFs can encode other non-structural proteins, including a 11 kDa protein and the less characterized 9.0 and 7.5 kDa proteins. The capsid forms an icosahedral structure in T = 1 arrangement, about 25 nm in diameter, composed of 5–10% VP1 and 90–95% VP2 proteins. It is resolved in its atomic structure for the capsid shell but not for the N-terminus of VP1 (VP1 unique region, VP1u) [4]. A schematic diagram of B19V genome organization is depicted in Figure 1.

## 3. The Lifecycle

**Figure 2:**
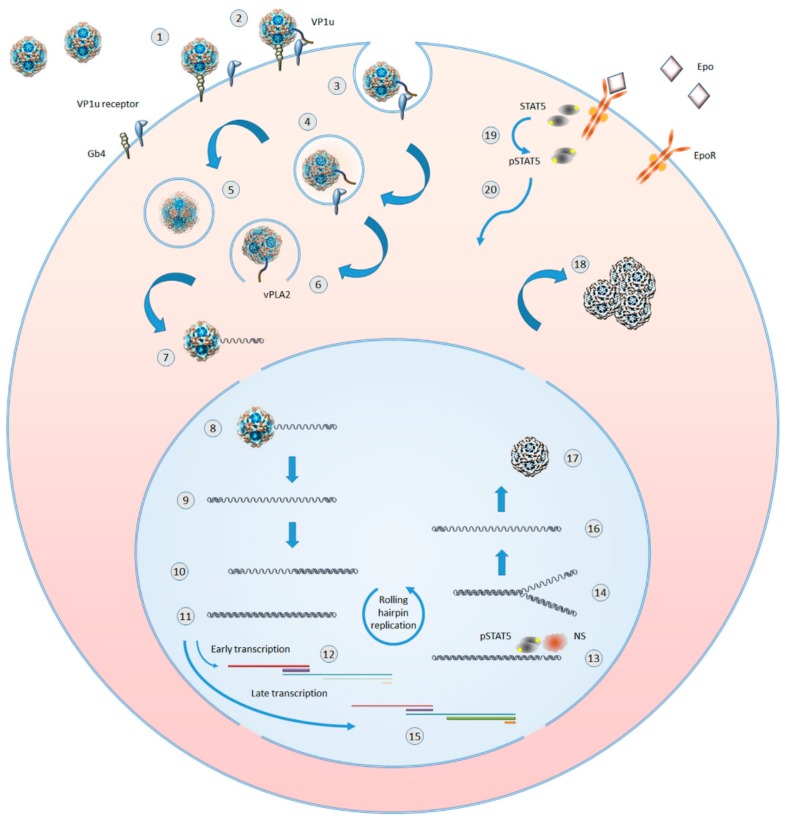
Outline of B19V replicative cycle in erythroid progenitor cells. 1: virion binding to globoside. 2: extrusion of VP1 unique (VP1u) region and binding to an erythroid specific receptor. 3: clathrin-mediated endocytosis. 4: virions in endosomal vesicles. 5: virion processing within endosomes. 6: VP1u-associated viral phospholipase (vPLA2) mediated virion escape from endosomes. 7: partial uncoating and externalization of viral ssDNA. 8: translocation in the nucleus and complete uncoating. 9: parental ssDNA and onset of macromolecular syntheses. 10: hairpin-primed second strand synthesis. 11: formation of dsDNA replicative intermediate. 12: early phase of transcription on the parental template, mainly of mRNAs for NS protein. 13: dsDNA nicked by NS and priming of replication in coordination with cellular proteins. 14: replication by a rolling hairpin mechanism, via self-primed single-strand displacement mechanisms. 15: late phase of transcription on the replicative intermediates, mainly of mRNAs for VP and 11kDa proteins. 16: progeny ssDNA released from the replicative intermediates. 17: incapsidation of progeny ssDNA molecules in newly formed virions. 18: accumulation of virions before their release via cell lysis or apoptosis. 19: Epo binding by Epo receptor (EpoR), EpoR activation, and STAT5 phosphorilation. 20: pSTAT translocation in the nucleus where it is essential for formation of a functional replicative complex.

B19V shows a selective tropism for cells in the erythroid lineage in the bone marrow, cells that are susceptible to viral infection and permissive for a productive replicative cycle depending on their differentiation stage and proliferation rate. Such tropism, and a productive outcome of infection, can be considered the result of a double adaptation of virus to a specific cell population. The first involves the recognition and binding to specialized receptors that define as target cells a restricted cell population with a high proliferative potential, namely erythroid progenitor cells and in particular cells at the proerythroblast differentiation stage. The second involves a strict dependence of viral replication to the cellular response to convergent lineage-specific physiological stimuli, such as Erythropoietin (Epo) pathway activation and hypoxia. A schematic diagram of B19V lifecycle is depicted in Figure 2.

Binding events involve domains on the viral capsid interacting with cellular receptors. An initial event is the interaction of the capsid shell with the membrane glycolipid globoside, which is present on erythroid progenitors, as well as on mature erythrocytes where it constitutes blood antigen P, but also on many other tissues mainly of mesodermic origin [6]. Since its first identification as a binding receptor [7], and the observation that its absence prevented infection in cells as well as individuals [8], subsequent reports presented contrasting evidence, either characterizing or questioning the binding of capsids to globoside as a necessary first step for cell infection [9,10,11]. However, even a transient binding of a capsid to globoside can trigger conformational modifications leading to exposure of the VP1u region [12], allowing interaction of its N-terminal region with a specific, but yet uncharacterized receptor whose distribution in cells of erythroid lineage matches the susceptibility of cells to productive infection [13,14,15]. Following binding, internalization via clathrin-mediated endocytosis can occur, the phospholipase activity associated to the VP1u region consents escape from the endosome, and by subsequent coordinated intracellular transport and uncoating events, a single-stranded genome is finally delivered in the nuclear environment [16,17].

In the nucleus, a series of macromolecular syntheses occurs leading to a productive replicative cycle [18,19]. On the single-stranded DNA template, cellular DNA repair synthesis generates a double-stranded DNA template that can serve for both transcription and replication of the viral genome. An early phase of transcription mainly produces mRNAs coding for the NS protein, which, acting together with cellular replicative machinery promote replication of the genome by a rolling hairpin mechanism. Replication is then followed by a late phase of transcription, mainly producing mRNAs coding for the structural VP and 11 kDa proteins. Accumulation of VP proteins eventually leads to the assembly of capsids, encapsidation of progeny single-stranded genomes, and release of virions from infected cells.

In the erythroid lineage, a productive viral replication and release of virus are restricted to differentiation stages ranging from colony forming unit-erythroid (CFU-E) to erythroblasts, indicating that both lineage- and differentiation-specific factors are necessarily involved in promoting viral macromolecular syntheses [20]. Viral replication is critically dependent on erythropoietin stimulation and is enhanced in hypoxic conditions [21,22], through a signaling cascade leading to formation of a functional replicative complex involving the viral NS in concert with cellular proteins, including the DNA replication polymerase δ and polymerase α [23]. A crucial role is exerted by phosphorylated STAT5 protein, which is a common terminal of Epo- and hypoxia-stimulated pathways [24]. A key event is the regulated switch from the early pattern of viral expression, characterized by transcription on the parental template mainly leading to NS protein production, to the late pattern of expression, with coordinated onset of DNA replication and enhanced transcription of the progeny templates leading to increased VP protein production [20]. In addition, the 11 kDa protein, expressed in the late phase, may also play a role in facilitating viral genome replication [25,26]. A deeper understanding of the mechanistic details of viral replication, including fine characterization of the molecular machinery and activation pathways involved, is in progress and will offer increasing opportunities to identify specific targets for the development of antiviral strategies.

In infected erythroid progenitors, the virus exerts a complex series of effects on the cellular environment, including induction of a DNA damage response, arrest of the cell cycle, and induction of apoptosis [27,28]. This cytotoxicity causes a temporary block in erythropoiesis and can lead to a transient or persistent erythroid aplasia. The interactions between the viral and cellular factors are still incompletely characterized, for example, in the possible activation of cellular sensors to viral infection, in the induction of cellular responses to restrict viral replication, or in priming of innate immunity. Therefore, in this subject area, a better understanding of the mechanistic details will probably offer opportunities to define novel antiviral strategies.

In addition to erythroid progenitors, the virus can also, although less efficiently, infect other cell types in diverse tissues. B Lymphocytes in tonsillar tissues have been shown to harbor the viral genome, and can be infected by an antibody-dependent uptake mechanism [29]. Endothelial cells constitute a diffuse cellular target susceptible to viral infection, also by an antibody-dependent mechanism [30]. The viral genome has progressively been detected in almost all solid tissues and organs, mostly in endothelial or stromal cells but occasionally also in parenchymal cells [31]. In non-erythroid tissues, infection is usually abortive, viral DNA can remain silent, and when transcription occurs this is normally at low levels. In these cases, transcription is mostly limited to early mRNAs—including those for the NS protein—and transcription of late mRNAs, including those for VP proteins, has been documented in tissues such as heart, liver, synovia, and skin. In these cells, a limited expression of viral proteins may contribute to pathological effects mainly by indirect mechanisms, such as modification of the cellular expression profile and the induction of inflammatory or autoimmune processes [31]. The frequent outcome is rather the persistence of the viral genome in tissues [32], probably in the episomal form although integration of the viral genome in the cellular genome of erythroid progenitor cells has been detected in an in vitro experimental system [33]. Reactivation, if it can happen, appears to be a sporadic event, not firmly documented in the literature. Notably, persistence of the viral genome in tissues appears to be lifelong and constitutes a repository of archived genomic sequences [34,35], also leading to exciting hints regarding the evolutionary history and genetic diversification within the species [36].

## 4. The Pathologies

B19V is a virus commonly diffuse in the population, and responsible for a wide spectrum of clinical manifestations (comprehensively reviewed in [2,3]). Following contact, normally through the respiratory route, the virus gains access to the circulation and reaches the bone marrow where it can infect erythroid progenitor cells. The pathogenic effects, typically in the form of pure red cell aplasia (PRCA), result from the capacity of the virus to induce cell-cycle arrest, block erythroid differentiation and proliferation, and eventually apoptosis of infected cells. The clinical impact on the host depends on the degree of inhibition of erythropoiesis, linked to the volume and turnover rate of the erythroid compartment, while the course of infection depends on the capacity of the immune system to mount an effective specific response [37].

In individuals with normal erythropoiesis and immune system response, bone marrow infection is limited in extent and temporal frame, is usually asymptomatic from hematological perspective, and is progressively cleared by the development of a neutralizing immune response. In the presence of an altered erythropoietic process and an expanded erythroid compartment, because of underlying genetic defects or stressed physiological conditions affecting the cellular turnover, infection can induce a more severe block in erythropoiesis, which usually manifests in the form of an acute episode of profound anemia. In the presence of defects of the immune system and a consequent inability to control, neutralize, and clear the virus, infection may become persistent and manifest with chronic anemia of different grades. Rarely, the infection has been linked to bone marrow necrosis [38], in addition to a wide variety of blood diseases and cytopenias of lineages other than the erythroid lineage [37], by mechanisms that still require investigation.

Productive replication in the bone marrow leads to a secondary viremic phase initially characterized by high viral load levels (up to 10^12^ virus/mL), followed by a systemic distribution of the virus and preluding to possible late clinical manifestations. In this later phase, both the virus and specific antibodies are present in the blood, so that immune-mediated inflammatory processes are mainly assumed to explain possible pathological processes. Different non-erythroid cell types, including endothelial, stromal, or synovial cells, can also be infected, and pathogenetic mechanisms directly related to the viral presence and activity can be hypothesized. However, only sporadically have some specific markers of viral activity been definitely localized within non-erythroid cells, and causally linked to pathological processes by viral-induced, usually proinflammatory, pathogenetic mechanisms [31].

Typically in this later phase, B19V infection is the cause of erythema infectiosum in children, and of arthropathies mainly in adult patients, with a tendency to chronicity. While B19V has been progressively detected and implicated in many clinical situations involving disparate tissues and organs, in particular it has been recognized as a relevant cardiotropic virus, responsible of acute myocarditis and possibly involved in the development of chronic cardiomyopathies (an intense debate recently summarized in [39]). B19V can be involved in the development of autoimmune disorders [40], and possible mechanisms involving epitope cross-reactivity [41] or the formation of apoptotic bodies induced by NS protein expression have been proposed [42,43].

B19V can cross the placenta and infect the fetus [44], where infection of erythroid progenitors can induce a block in fetal erythropoiesis whose effect will depend on the fetal developmental stage, the rate of expansion of the fetal erythroid compartment, and the maturity and efficacy of both maternal and fetal immune response. The virus can be detected in erythroid progenitor cells, located in liver and/or bone marrow depending on the gestational age, in erythroid cells circulating in the vessels of several tissues, in endothelial placental cells [45], and in the amniotic fluid [46]. Transplacental transmission can occur in about 30–50% of cases, and lead to fetal hydrops and/or fetal death in ~10% of cases (comprehensively reviewed in [47,48]).

## 5. Need for Treatment and Current Options

Although most infections are mild and self-limiting, there are situations where B19V infection can be severe and lead to the need of clinical care. These include hematological complications, from transient aplastic crisis to chronic pure red cell aplasia, to rarer clinical presentations involving bone marrow necrosis or autoimmune-mediated hematological disorders [37]. The role of B19V in acute or chronic myocarditis, although debated, is of relevance [39]. More classical manifestations such as arthropathies can chronicize and be invalidating to patients for extended time periods [40], and even dermatological manifestations can be atypical, severe, and lead to hospitalization [49]. Intrauterine transmission can severely affect the fetus, possibly leading to fetal death, development of fetal hydrops, and in rare cases congenital infection [44,47,48].

The diverse clinical presentations first of all call for an appropriate diagnostic approach [5]. B19V infections should be investigated not as a rare entity, but as a frequent possibility, especially in the context of peaks of incidence. Molecular and immunological diagnostic assays are now widely available and their rational use can lead to a prompt diagnosis and to appropriate clinical management. The clinical attitude towards B19V infection is normally conservative, in the idea that infection is self-limiting, and that the development of a specific immune response as measured by the production of specific and neutralizing antibodies will be effective in controlling the virus. However, this is not always the case. Acute infections can be clinically severe while an impaired immune response can lead to persistent infections. When required, supportive or symptomatic treatments can be used. Blood transfusions are required to overcome acute or chronic anemia, nonsteroidal anti-inflammatory drugs are generally used although with limited efficacy to relieve inflammatory symptoms in cases of arthritis and arthralgias, while scattered case reports suggest the utility of corticosteroids in cases of atypical inflammatory presentations. The management of intrauterine infections is also conservative, and when fetal Hb levels fall below a clinically defined threshold as measured by non-invasive Doppler ultrasonography determination of middle cerebral artery peak systolic velocity, it can rely with good success rates on intrauterine transfusions [50].

The gap in the development of antiviral strategies and in particular the availability of antiviral drugs directed against B19V as compared to other viruses is striking [51]. A vaccine against B19V is an attainable goal, technically feasible, composed of VLPs produced in heterologous expression systems, and following progressive development [52,53,54] now shows promising characteristics in terms of immunogenicity and absence of reactogenicity [55,56]. However, because of the lack of relevant animal models, it is still at the very beginning of clinical evaluation, and its implementation is not included among the WHO priorities. Administration of high doses of intravenous immunoglobulins (IVIG) is presently considered the only available option to neutralize infectious virus and mainly finds indication to control infections in cases of an impaired immune system response [57,58,59]. The beneficial effects of IVIG treatments are recognized, even if high-doses and repeated cycles may be required, and it is considered that IVIG are not sufficient to resolve infection unless a patient’s own antiviral immune response develops and becomes effective.

Active research in the development and refinement of antiviral strategies directed against B19V should be considered of the highest relevance. In addition to the use of IVIG, the discovery of antiviral drugs with significant activity against B19V would offer important opportunities in the treatment and management of severe clinical manifestations. In particular, these would include the treatment of severe hematological complications in the acute phase of the infection, especially in subjects with stressed erythropoiesis, or the treatment of chronic infections in case of deficits of the immune system. Furthermore, antiviral compounds might be used in the implementation of prophylactic treatments, for example to reduce the risk of infection in immunosuppressed individuals as part of a general preventive or pre-emptive approach.

## 6. Passive Immunization

Administration of IVIG is currently the indicated treatment when patients are in need of controlling B19V infections, in the case of chronic infections or more rarely in acute infections with clinical severity, and inability of mounting an efficient immune response. Several accepted guidelines suggest cycles of 2 g/kg in 5-days courses, to be repeated if unsuccessful, but studies have not been carried out to determine an optimal therapeutic scheme. Efficacy of IVIG treatment has been assumed more on circumstantial and empirical evidence than on high-quality evidence-based assessments [60]. Available data obtained from small case series and literature reviews indicate that IVIG treatments are effective with good success rates [61], but IVIG treatments are likely to be underreported in the literature, and this more so in the case of failure.

The mechanism of action of IVIG is also not fully investigated. Possibilities include capacity of inhibiting the virus by direct binding of specific anti-B19 Ig, normally present in IVIG preparations, to functionally relevant epitopes on the viral capsid, thus preventing infectivity. However, in cellular models, binding and penetration steps may not be inhibited, while the successive phases of macromolecular synthesis can be severely impaired, both at transcriptional and replicative levels [62]. Possibly, binding to antibodies prevents the virions from correct intracellular trafficking, uncoating, and translocation of viral genome in the nucleus. In general, IVIGs may also exert their effect via immune modulatory mechanisms [63], and this might contribute to their efficacy, while the possibility exists that in peculiar situations the immune complex formation exacerbates inflammatory stimuli. A peculiar case came from the experience of treating the B19V-related chronic fatigue syndrome, with reports of successful treatments [64] as well as paradoxical response [65].

An alternative to IVIG would be the use of human/humanized monoclonal antibodies specifically targeted to B19V, as more and more are available for other infectious agents. This approach showed promising results in an early initial report [66], but would require further research to become an available option. In this case, a neat definition of relevant neutralizing epitopes is required. It is known from studies in the general population that antibodies recognize largely VP2 conformational antigens coupled to VP1u region linear antigens [67,68]. Neutralizing epitopes are distributed along most of the VP2 protein and in the N-terminal region of the VP1u [69,70,71]. A comprehensive epitope mapping on the capsid shell surface is still to be obtained, but recently a first structure of a parvovirus B19 capsid complexed to antigen-binding fragments (Fabs) from a human antibody has been obtained by cryo-electron microscopy (cryo-EM), showing binding to a quaternary structure epitope formed by residues from three neighboring VP2 capsid proteins [72]. The structure and location of VP1u is not determined, but it can be observed that the immunogenic region corresponds to the receptor-binding moiety essential for virus infectivity [73].

## 7. The Quest for Antiviral Agents

So far, some factors have been critically limiting in the search for compounds with antiviral activity against B19V. The virus requires demanding cell culture conditions and in vitro infections show a restrictive pattern with relatively low productivity, thus most experiments need to rely on the availability of the native virus obtained from viremic patients. Research in the field still offers an incomplete characterization of the viral lifecycle, of the viral proteome, and of the molecular machinery coopted to viral replication. The first problem limits the feasibility of a high-throughput screening against available chemical libraries, the second has until now hampered the rational design of specifically targeted drugs. To overcome these barriers, standardized model cell cultures and infectivity assays are required in the first instance. Then, these studies can also take advantage of the availability of cloned viral genomes that possess replicative competence and the ability to yield infectious viruses in standardized conditions.

Mainly, two cellular systems can be used to support viral replication in vitro and study the antiviral activity of tested compounds: primary erythroid progenitor cells (EPCs) and the cell line UT7/EpoS1. EPCs are primary cells that more closely resemble the natural target cells within the bone marrow environment [74]. EPCs obtained from peripheral blood can be cultured in conditions that promote proliferation and differentiation along the erythroid lineage [75], and progressively become permissive to viral replication, mostly at the proerythroblast stage [20]. However, in vitro culture conditions only approximate conditions in the bone marrow environment, and EPCs constitute a heterogeneous population with respect to the differentiation stage, proliferation rate, and metabolic activity. UT7/EpoS1 is a cell line of myeloblastoid origin, the most permissive and commonly used for B19V [76]. Although permissiveness is restricted to only a subset of cells [77], in these, the degree of replication of viral DNA is comparable to EPCs [78,79]. Both EPCs and UT7/EpoS1 cells require Epo stimulation to support viral replication. EPCs and UT7/EpoS1 cells thus provide manageable and appropriate models for investigating compounds with antiviral activity against B19V.

For infection, native virus obtained from viremic serum samples is normally used, with limitations implied due to both the limited availability and the unpredictable variation inherent in the use of individual clinical isolates within a heterogeneous biological matrix such as plasma or serum. The possibility of obtaining virus with complete biological activity starting from cloned DNA templates has been explored. Clone pM20 was established in 2004, has been shown to possess replicative activity, and it has been mainly used in transfection experiments [80]. More recently, functionally competent clones have been constructed starting from a synthetic consensus sequence (named EC01), possessing both replicative activity following transfection and the ability to yield infectious virus at high titers following serial amplification passages in EPCs [81]. In perspective, a crucial advantage of using virus obtained from cloned genomes is the possibility of conducing direct mutagenesis and sequence-function correlation studies to define targets relevant for antivirals.

In the experimental setup, the antiviral effects of tested compounds can be accurately evaluated by qPCR-based assays, to measure the variation in the abundance of viral DNA or mRNAs following a course of infection [78,79]. In situ hybridization (FISH) assays for viral nucleic acids or immunological (IIF) detection of viral proteins, can be used to measure variations in the frequency of productively infected cells [77]. Then, the extent of inhibition of viral replication exerted by the compounds can be determined by standard dose-response curve to yield the EC_50_ values. Concomitant effects of tested compounds on cell viability and cell proliferations rates need to be determined, usually by standard formazan-based, or equivalent assays, and by BrdU incorporation assays, to yield CC_50_ values. Selectivity indexes can then be calculated to assess the specificity of action of a tested compound.

Within this experimental frame, research can be aimed at the discovery of antiviral compounds targeted to crucial functions within the viral lifecycle. Antiviral compounds can be intended for conditioning the cellular environment as non-permissive, or specifically targeted to viral proteins as direct antiviral agents. Recent work in this field led to the first identification of compounds with antiviral activity against B19V. Alternative approaches to antiviral discovery have been followed until now: a strategy based on drug repositioning; a strategy based on investigation of known antiviral compounds for a possible activity against B19V; a serendipity approach in screening small chemical libraries of compounds with possible antiviral activity; and a search for direct antiviral compounds by targeted biochemical screening. The first approach yielded antiviral activity provided by the cell-proliferation inhibitor hydroxyurea (HU) [82], also used as a disease-modifying drug in the treatment of sickle cell disease. The second approach first yielded the acyclic nucleoside phosphonate cidofovir (CDV) [83,84], though with suboptimal activity, and then its lipid conjugate brincidofovir (BCV) [85], with substantially enhanced activity. By the third approach, a few coumarin derivatives showed promising characteristics [86]. In the search for direct antiviral agents, identification of a key function of viral NS protein allowed screening of a small molecule library by which a few compounds showed inhibitory activity, which were then assessed for inhibition of viral replication in a cell-based assay [87]. A summary of the results is reported in Table 1.

### 7.1. Hydroxyurea

Hydroxyurea (HU) is an inhibitor of DNA synthesis targeting cellular ribonucleotide reductase enzyme [88]. HU behaves as a ‘virostatic’ antiviral agent in combined therapy with deoxynucleoside analogs, with the assumption that HU depletes the intracellular deoxyribonucleotide pools required for viral replication, enhancing deoxynucleoside analogs incorporation [89,90,91,92]. Of relevance, the drug finds indication in the therapy for sickle cell disease (SCD) in adults [93], and increasingly in the pediatric SCD population [94], in which B19V infection is a major cause of severe complications.

Experimentally, HU demonstrated a measurable inhibitory effect on B19V replication in both EPCs and UT7/EpoS1 cells [82]. Complete inhibition of viral replication was obtained at >1 mM, and observed EC_50_ values were 96.2 µM and 147.1 µM in UT7/EpoS1 and EPCs, respectively. Cellular DNA replication was also affected with HU concentrations leading to a 50% reduction of DNA synthesis at 706.9 µM and 494.0 µM in UT7/EpoS1 and EPCs, respectively. A cytostatic effect was confirmed in both systems, with a 50% reduction of viability observed at 581.9 µM for UT7/EpoS1, and 584.8 µM for EPCs. The observed reduction in cell viability could be ascribed to the cytostatic effect of the drug rather to a cytotoxic effect related to loss of membrane integrity.

The effective concentrations of HU inhibiting B19V replication are comparable with those obtained for other human pathogenic viruses [89,90,91,92], and lower than those interfering with cell proliferation. In both cell systems, HU confirmed its cytostatic and ‘virostatic’ effects, in agreement with its inhibitory activity on cellular ribonucleotide reductase [88]. In EPCs, the variation in the distribution of cellular differentiation markers indicated an inhibitory effect on the differentiation of cell population and a reduction in the generation of more mature cells, exerted by both HU and virus with additive effects. HU prevented cells from leaving the G1/S phases with a related reduction of cells in G2/M, in both infected and uninfected cells. EPCs arrested with a 2N DNA content may be not competent to engage in B19V active replication, thus contributing to the antiviral effect of HU together with the lowering deoxyribonucleotide levels within cells.

As mentioned, HU is used as a disease-modifying drug in sickle cell disease, where it can exert a protective effect due to the inhibition of erythroid cell proliferation/differentiation [93,94]. SCD is a typical situation where infection with B19V can exert profound pathological effects, requiring hospitalization and intense supportive therapy, so a dual effect of the compound both on the course of the underlying disease and on the course of infection by its antiviral activity would be beneficial. In treated SCD patients, HU can reach peak plasma concentrations of ~250–400 mM [95], indicating that HU levels sufficient to reduce B19V replication in vitro are achievable in vivo. A survey of clinical records of SCD patients undergoing HU therapy showed indeed a protective effect of HU against B19V infection compared to non-treated patients, at least in terms of severity of disease and need for treatments [96]. As determination of peak viremic levels in the two group of patients were not presented, there are still two hypotheses to explain this observation, either that such protective effect is due to a lower viral replicative activity consequence of the antiviral activity of HU, and/or it can be indirectly linked to a prolonged lifespan of erythrocytes. Further clinical investigation would be helpful to explore the potential beneficial clinical effects of HU.

### 7.2. Nucleotide Analogues: Cidofovir (CDV) and Brincidofovir (BCV)

A different strategy involved the evaluation of broad-spectrum antiviral compounds for a possible activity against B19V. In particular, the acyclic nucleoside phosphonate cidofovir (CDV) has shown activity against all families of human, not retro-transcribing dsDNA viruses [51,97,98], including viruses not encoding their own DNA polymerase. B19V is a ssDNA virus, but depending for its replication on cellular DNA polymerase activity acting on a dsDNA replicative intermediate, so potentially inhibited by a nucleotide analogue incorporated in a nascent DNA molecule. Experimental results confirmed this working hypothesis [83,84].

In UT7/EpoS1 cells, CDV exerted a measurable inhibitory effect on B19V replication in the range 0–500 µM, achieving complete inhibition of viral replication at the higher concentration and EC_50_ values in the range 7.45–41.27 µM (depending on the multiplicity of infection, in the range 10^1^ to 10^4^ geq/cell). Viral transcription was less affected by the presence of CDV, with a significant reduction only at the higher concentration tested, coupled with a block in the shift from an early to late pattern of transcription possibly correlated to the block in replicative activity. Concurrently, a progressive reduction in the number of FISH and IIF positive cells was observed with increasing the concentration of CDV. In these cells, CDV did not alter cell viability or proliferation to a statistically significant extent.

The inhibitory activity of CDV was much less relevant in EPCs. A statistically significant effect of CDV on viral DNA replication was evident only for the 500 µM concentration, and even at this highest concentration inhibition was not complete, but only in the range 68.2–92.8% depending on the multiplicity of infection. CDV added to cell cultures did not alter EPCs viability or proliferation to a statistically significant extent. These results suggest that the cellular environment is crucial to the activity of CDV, and different hypotheses to explain such dependency include a slower uptake of CDV within EPCs, a slower metabolic activity with reduced production of the active metabolite, CDV diphosphate (CDV-PP), or a difference in the replicative machinery involved in replication of viral genome with different sensitivity to CDV.

In EPCs, addition of CDV led to a decrease in the release of virus in the supernatant of cell cultures, and in a reduction of its infectivity in subsequent rounds of infection. At the highest multiplicity of infection tested, the overall reduction in virus yield and infectivity was >90%, a result arising as the additive effect of the inhibition of replication within EPCs, with a lower production of infectious virions (68–70% reduction), and a reduced replicative activity of viral DNA (75% reduction). It is possible to hypothesize that incorporation of CDV in progeny DNA strands is responsible for the reduced replicative activity observed.

The hypothesis that prolonged incubation with CDV could lead to a more profound inhibition in viral replication was tested by assessing at the highest multiplicity of infection and CDV concentration: (i) the effect of preincubation of cells with CDV prior to infection; (ii) the effect of an extended time course of infection in the presence of CDV; and (iii) the effect of serial passage of virus under antiviral pressure exerted by CDV. Reduction in viral replicative activity was observed in all of these situations. Preincubation with CDV reduced viral replication more than 90%, significantly higher than addition of CDV following infection. Extended incubation with CDV had modest effects on cell viability, although a reduction in cell proliferation up to 70% was observed, but inhibition of viral replication was observed at about 80%, coupled to a similar reduction in the amount of virus released in the cell culture supernatant. An overall reduction in viral replication higher than 96% was finally observed following three serial passages of virus in EPCs in the presence of CDV, suggesting that a constant pharmacological pressure exerted by CDV can alter the viral replication dynamics to a significant extent.

Overall, these results firstly indicated that inhibition of B19V replication could be achieved by effect of an antiviral agent, but the efficacy of CDV appeared critically dependent on the cellular environment. In primary EPCs, the inhibitory activity of CDV was significant only at the higher concentration tested and by extended exposure, which is impractical in clinical terms. Moreover, concerns of toxicity on CDV prevent its widespread use and would be a major obstacle in developing an effective treatment option for B19V, prompting for further research. Thus, in a development aimed at overcoming these limits, the antiviral activity of Brincidofovir (BCV) was further evaluated [85]. BCV (CMX001) is a modified form of CDV, where the acyclic nucleotide phosphonate has been conjugated to a lipid moiety, with the result of a more potent activity demonstrated against dsDNA viruses, better bioavailability and absence of toxicity [99,100,101].

Experimental investigation confirmed an enhanced antiviral activity of BCV compared to CDV in both cellular systems, and in particular in EPCs only BCV but not CDV yielded complete inhibition of viral replication [85]. For BCV, EC_50_ values were in the range 6.6–14.3 µM in EPCs and 0.22–0.63 µM in UT7/EpoS1 cells. In comparison, EC_50_ values for CDV were >300 µM in EPCs and 16.1 µM in UT7/EpoS1 cells. Accordingly, effects on cell viability were observed for BCV as opposed for CDV, with calculated CC_50_ values in the range 93.4–102.9 µM in EPCs and 59.9–66.8 µM in UT7/EpoS1. Specificity in the antiviral effect was confirmed by comparing the activity of the two enantiomeric forms of BCV, (S) and (R), where only BCV (S) and not BCV (R) is the active enantiomer. BCV (S) utilizes the lipid uptake pathway in cells, leading to an increase in the effective concentration of the active antiviral, CDV-PP, and therefore to enhanced antiviral potency [102]. In EPCs, the selectivity index values for BCV (S) and BCV (R), determined as the ratio CC_50_/EC_50_, were 6.5 and 1.6 respectively, yielding an S/R ratio of 4.0, and in UT7/EpoS1, SI values were 95.1 and 1.3, yielding an S/R ratio of 73.2. Such high S/R ratios indicate an active and specific antiviral role of CDV-PP, derived from BCV (S), as opposed to a nonspecific cytotoxic effect indirectly causing inhibition of viral replication.

As a common mechanism of action, the antiviral activity exerted by both BCV and CDV is due to CDV-diphosphate (CDV-PP) which is used as an alternate substrate for viral DNA synthesis [103]. Both BCV and CDV are broad-spectrum antivirals that possess activity against all dsDNA viruses, including those that do not encode their own polymerases [104,105,106], even though the mechanism of inhibition has not been fully explained in this case [107,108]. By also showing antiviral activity against B19V, the spectrum of activity of CDV-PP is thus expanded to include a ssDNA virus, even if it should be considered that the B19V genome replicative intermediates are actually dsDNA forms that utilize the host replication machinery and metabolic environment. The exact mechanism(s) of action of both CDV and BCV in the inhibition of B19V replication, and its dependence on the different cellular environments, warrants further investigation.

The identification of broad-spectrum antiviral compounds with in vitro demonstrated antiviral activity also including B19V might be considered of relevance as a rationale for evaluating the use of these drugs in the treatment of patients. Use of CDV is not recommended, but BCV proved effective in inhibiting B19V replication at concentrations that are attainable in vivo [109], has a known safety profile [110] and compares favorably with other antivirals in its activity against dsDNA viruses [111], thus opening the possibility of its use also to treat B19V infections, given a cautious approach required for clinical management.

### 7.3. Serendipity Approach: Coumarin Derivatives

As discussed, a high-throughput screen against available chemical libraries is hardly achievable, but a more targeted serendipity approach in the screening of small-scale chemical libraries is a practicable option. Small libraries might include compounds of different chemical nature, selected on the basis of known and potentially relevant biological activities, and can be investigated by using established biological and analytical protocols.

By this approach, an initial screening of a small chemical library was carried out indicating some coumarin derivatives as scaffold molecules with promising activity against B19V [86]. Coumarins are already in use as therapeutic agents in humans [112] and some products characterized by a coumarin nucleus are known to possess some antiviral activity in many model systems [113], although their precise mechanisms of action is unknown. Following the initial screening that led to identification of a compound with promising characteristics (3-(imidazo[2,1-b]thiazol-6-yl)-2H-chromen-2-one), serial chemical modifications of this molecular scaffold yielded a derived small chemical library that was further evaluated, leading to additional identification of differently substituted molecules with measurable specific antiviral activity. Overall, by testing these compounds at their highest attainable concentrations, inhibition of viral replication in both EPCs and UT7/EpoS1 was only partial, in the range 60–82% in the best case and mainly inUT7/Epos1 cells. Effects on cell viability were also relevant, with inhibition up to 30–40% in the worst case. However, for three of these compounds, prevalent antiviral compared to cytotoxic effects was observed, with SI > 2.4–4.0. Furthermore, in UT7/EpoS1 cells, the inhibition of viral replication followed a dose-response curve (calculated EC_50_ values ~6.4–6.7 µM) that was significantly different compared with a rather unspecific effect on cellular viability, suggesting that the mechanisms for the observed activity of these compounds could either involve inhibition of a viral target or of a cellular function specifically needed by the virus during its replicative cycle.

While such serendipity approach allows small-scale screening and can yield appreciable results, it is a laborious approach that can be hardly rewarding in terms of success. Even when screening molecules with promising characteristics, the hit rate is unpredictable, and in the presence of any antiviral activity the identification of the target, whether cellular or viral, is an endeavoring task. Nonetheless, by following this approach, characterization of both target and mechanisms of action would be required, not least due to the need to refine the design and synthesis of molecules better suited to exert their antiviral activity.

### 7.4. Direct Antiviral Agents

A different experimental approach can be followed in the search for direct antiviral agents. Detailed knowledge of the viral lifecycle and of mechanistic details of the molecular machinery involved, can allow the identification of critical targets, whose inhibition would prevent viral replication or at least viral cytotoxic effects. By this approach, functions crucial to the viral lifecycle should be attributed to specific genes, gene regions or even protein domains. In vitro assays, also using recombinant proteins, should be developed to measure correlate activities, so that any inhibition of these activities by tested compounds might be investigated firstly in screening experiments using biochemical assays, and then in cell-based assays. Such an approach has been reported in the literature, in the search for compounds that are able to inhibit the endonuclease activity associated with viral NS protein [87].

Within the B19V proteome, the NS protein is synthesized in the early phases and exerts crucial functions during the viral lifecycle [19]. The protein is involved in the terminal resolution reaction, which is essential for the rolling hairpin replication of viral DNA, and both an endonuclease and helicase activity are required, mapped to distinct domains of the protein. It has an activating role on the viral promoter as well as a heterologous trans-activating action on several cellular promoters, this activity is mapped to a different protein domain. The protein is responsible for interactions with cellular pathways, including the induction of a DNA damage response, dysregulation of cell cycle, and apoptosis. Since crystallographic studies related to the NS protein have not been produced, its molecular structure can be only predicted by analogy to other replicative molecules in the family, for example, rep proteins of Adeno-Associated Virus (AAV), but to an approximation that impairs the rational design of ligands and inhibitory molecules. The functional mapping of the NS protein indicates the presence of different domains and associated functions, localizes putative active sites, and allows the design of biochemical assays to measure functional activity.

The N-terminus (aa. 2–176) of NS1 possesses DNA binding and endonuclease activity, and an endonuclease motif resides between amino acids 137 and 145 [114]. The presence of a functional endonuclease domain is necessary for replication of the viral genome, by allowing terminal resolution and continuing rolling hairpin replication. This function can be monitored also by in vitro biochemical assays, by using a purified recombinant protein fragment obtained from a prokaryotic expression system and measuring activity by the amount of cleaved oligonucleotides of appropriate target sequence [115]. This biochemical assay, further improved using a fluorophore-based reporter system, proved suitable to evaluate a specific inhibitory activity of tested compound in a direct target-based assay [87]. The convenient assay format allowed screening of a selected chemical library and led to the identification of a subset of compounds with significant (>80%) in vitro endonuclease-inhibiting activity at concentrations <10 µM. Among these, three compounds of flavonoid-like structure were selected and further tested to determine their in vitro activity, in inhibiting the endonuclease nicking reaction, and in vivo to selectively inhibit B19V replication.

In vitro, dose-response curves showed IC_50_ values in the range 1.1–3.1 µM, making these compounds highly promising for subsequent evaluation in cell-based assays. In cell-based assays, involving both UT7/EpoS1 and EPCs, the selected compounds also showed a capacity to inhibit B19V replication as a dose- and time-dependent response, as determined by a cytometric assay to determine the fraction of productively infected cells. However, the inhibition of viral replication was only partial if not at the highest concentrations tested, at the expense of effects on cell viability. In UT7/EpoS1 cells, reported EC_50_ values were in the range 44.2–55.1 µM, compared to CC_50_ values in the range 180.9–227.0 µM, resulting in selectivity indexes of 3.3–4.4. In EPCs, EC_50_ values were in the range 33.5–53.9 µM, compared to CC_50_ values in the range 55.9–89.8 µM, resulting in selectivity indexes of 1.5–1.8. qPCR assay in EPCs following a 48 h infection course, the EC_50_ values were in the range 20.5–38.4, thus giving a selectively index in the range 2.4–2.7. Replication of the viral genome was already inhibited at an early time point post-infection, thus corroborating inhibition of the endonuclease function of the NS protein as a likely mechanism of antiviral activity.

Results of these studies, therefore, on one hand indicate that a target-oriented biochemical assay is feasible and useful, and that in principle it might be extended to investigate other viral functions crucial to the viral lifecycle. On the other hand, these results clearly indicate a major problem arising when considering the whole virus–cell system as a target, as opposed to isolated functions of protein domains. Issues of transport of compounds within cells, the metabolic fate of compounds, the accessibility of active sites in the context of a macromolecular assembly, and overall metabolic differences in cells, depending also on cell type, differentiation stage, proliferation rate, and metabolic activity, can all impact the efficacy of otherwise promising compounds in a crucial way.

### 7.5. Other Compounds

In the literature, a few other molecules have been reported, which possess measurable selective inhibitory activity on B19V or effects on B19V-infected cells. The assembly of a macromolecular machinery in the origin of replication of the viral genome requires not only a functional viral NS protein, but also cellular partners [23], including the phosphorylated form of STAT5, as a common terminus of the activation pathway triggered by Epo and enhanced by hypoxia [24]. The molecule pimozide is a known inhibitor of STAT5 phosphorylation, and accordingly it exerts an inhibitory effect on B19V replication, with only a minor effect on cell viability as determined by a colony formation inhibition assay. Pimozide is approved for use as an antipsychotic drug, so a dual use of the molecule might be conceivable but hardly proposed in the absence of aimed clinical studies.

The nucleotide analogue telbivudine, inhibitor of HBV reverse transcriptase, has been evaluated for its effects in B19V-infected endothelial progenitor cells, in the context of a pathogenetic mechanism proposed for B19V-induced endothelial disfunction and development of dilated inflammatory cardiomyopathy [116,117]. While telbivudine did not show any direct inhibitory effect on B19V replication or expression [116], it protected B19V infected endothelial progenitors by B19V-induced apoptosis [117], in a mechanism that possibly reverses B19V-induced dysregulation of BIRC3, thus intervening the apoptosis pathway and protecting susceptible cells from cell death.

Finally, the possible protective effects of IFN-beta have been evaluated in limited clinical trials for the treatment of dilated cardiomyopathies of supposed viral trigger, including B19V, considering its high prevalence in myocardial tissues, reporting promising results [118,119,120]. The interactions of B19V and the innate immune system are not yet well characterized, either in terms of sensor activation, induction of effector mechanism, and possible viral escape strategies. A better understanding of this aspect of virus–cell interaction will likely indicate novel antiviral strategies to pursue.

## 8. Conclusions and Perspectives

In conclusion, a few statements can be proposed. First, B19V infection should not be overlooked in clinical terms, and diagnostic uncertainties can be easily resolved when a correct diagnostic approach is followed. Thereafter, even if most infections do not require treatment, the management of more complicated cases would be advantageous with respect to the availability of dedicated treatments that incorporate both specific and unspecific antiviral agents, such as IVIG.

In the research on antivirals against B19V, some work has been done in recent years to close the gap with other viruses, first yielding compounds that are already in use and that could be retargeted to B19V. In the case of Hydroxyurea, available data already consent some clinical considerations, since continuous HU treatment is already used in SCD subjects that are at high risk of disease for B19V infection, and retrospective clinical data show a measurable protective effect against severe hematological manifestations. In the case of nucleotide analogues, these have been used, or are being investigated, for viral infections other than B19V, so that their safety profiles are known. While CDV is not used because of its toxic side effects, BCV is qualified as a promising broad-range antiviral. Given its demonstrated in vitro activity, its possible use in off-label situations justified by severe and non-responsive B19V infection might offer a clue to its efficacy in in vivo situations. Furthermore, its possible use as a broad-range antiviral and prophylactic agent in immunosuppressed individuals would also offer the opportunity to evaluate its efficacy in preventing or controlling B19V infections in the follow-up of these subjects. Research on targeted direct antiviral agents, on the other hand, might offer better-suited molecules, but will need to face evaluation of clinical safety coupled to efficacy, a demanding task in many respects.

In this context, continuing research in the field, with an ever-increasing knowledge regarding the viral lifecycle, the molecular machinery involved, and a precise understanding of virus–cell interactions, will offer novel opportunities for developing more efficient, targeted antiviral agents, that can be translated to available therapeutic options in the near future.

## Figures and Tables

**Table 1:** Compounds with reported activity against B19V.

Compound/Cells	Max Inhibition	EC_50_ (µM [CI])	CC_50_ (µM [CI])	SI (CC_50_/EC_50_)	References and Notes
HU					Ref [82]
UT7/EpoS1	100% at >1000 µM	96.2 [81.5–118.1]	581.9 [426.5–812.3]	6.0	moi in the range 10^1^–10^4^
EPCs	100% at >1000 µM	147.1 [121.4–190.4]	584.8 [478.5–722.3]	4.0	moi in the range 10^1^–10^4^
CDV					Ref [83,85]
UT7/EpoS1	100% at 500 µM	16.1 [12.9–20.2]	>500	ND	moi in the range 10^1^–10^4^
EPCs	ND	320.5 [173.9–590.7]	>500	ND	moi in the range 10^1^–10^4^
BCV					Ref [85]
UT7/EpoS1	100% at >10 µM	0.22 [0.19–0.25]	66.8 [62.0–72.9]	303.6	moi in the range 10^1^–10^4^
EPCs	100% at >100 µM	9.35 [8.84–9.89]	102.9 [87.6–120.8]	11.0	moi in the range 10^1^–10^4^
BCV (S)					Ref [85]
UT7/EpoS1	100% at >10 µM	0.63 [0.58–0.68]	59.9 [52.7–68.1]	95.1	moi 10^4^
EPCs	100% at >100 µM	14.3 [11.8–17.3]	93.4 [68.9–126.6]	6.5	moi 10^4^
BCV (R)					Ref [85]
UT7/EpoS1	100% at >100 µM	54.7 [42.7–69.9]	72.1 [62.5–83.1]	1.3	moi 10^4^
EPCs	100% at >500 µM	93.0 [77.4–111.8]	146.2 [106.4–200]	1.6	moi 10^4^
Coumarin derivatives					Ref [86]
UT7/EpoS1	82.1 at 12.5 µM	6.7	ND	4.0 at 12.5 µM	Compound #7; moi 10^4^
EPCs	65.0 at 12.5 µM	ND	ND	2.7 at 12.5 µM	Compound #8; moi 10^4^
Flavonoid compounds					Ref [87]
UT7/EpoS1					Following transfection (IIF)
Compound #7	NR	44.2 [25.6–62.8]	194.0 [171.0–216.3]	4.4	
Compound #135	NR	61.1 [60.8–61.4]	227.0 [206.0–248.0]	3.7	
Compound #201	NR	55.1 [47.2–63.0]	180.9 [160.4–201.4]	3.3	
EPCs					Following infection (IIF)
Compound #7	NR	37.6 [34.0–41.2]	55.9 [53.8–58.0]	1.5	
Compound #135	NR	53.9 [46.8–61.0]	89.8 [82.0–97.6]	1.7	
Compound #201	NR	33.5 [32.1–34.9]	60.0 [57.6–62.4]	1.8	

HU: hydroxyurea. CDV: cidofovir. BCV: brincidofovir. BCV (S), BCV (R): brincidofovir enantiomers S and R. SI: Selectivity Index. Moi: multiplicity of infection (genome copies/cell). ND: not determined. NR: not reported.
